# A new hybrid model for enhancing low-dose CT images using EfficientNetV2 and WGAN-GP: a multi-loss approach

**DOI:** 10.1186/s40001-025-03579-z

**Published:** 2025-12-09

**Authors:** Mohammad Hojjat, Mohammad Javad Shayegan

**Affiliations:** Faculty of Engineering, Department of Computer Engineering, University of Science and Culture, Tehran, Iran

**Keywords:** EfficientNetV2-M, Low dose CT, Medical imaging, WGAN-GP, Multi-loss function

## Abstract

**Background:**

Low-dose computed tomography (LDCT) is widely used for medical imaging due to its reduced radiation exposure. However, LDCT images often suffer from significant noise, which can compromise diagnostic accuracy. This study aims to develop an effective denoising method that preserves critical anatomical structures while reducing noise, using a deep learning approach.

**Methods:**

We propose a novel LDCT image denoising method that integrates EfficientNetV2-M as a multi-scale feature extractor with a Wasserstein generative adversarial network with gradient penalty (WGAN-GP). The EfficientNetV2-M backbone (54.1 M parameters, depth scaling 1.2) employs seven stages of MBConv blocks with expansion ratios from 1 to 6, extracting hierarchical features at stages 3, 5, and 7. The model is optimized using three weighted loss functions: adversarial loss (Wasserstein distance), pixel-wise L1 loss (*λ*₂ = 1.0), and perceptual loss (*λ*₃ = 0.1). The discriminator employs gradient penalty with coefficient *λ* = 10 for training stability. Training employed 64 × 64 patches with batch size 128, Adam optimizer (learning rate: 1e-5) on the AAPM-Mayo Dataset. Image quality was assessed using peak signal-to-noise ratio (PSNR) and structural similarity index measure (SSIM).

**Results:**

The proposed method achieved a PSNR of 33.24 ± 0.15 dB and an SSIM of 0.92 ± 0.005 on the AAPM-Mayo Dataset across 10 independent runs, representing improvements of 4.0 dB and 0.04 over baseline LDCT images. Inference speed reached 12.5 FPS (0.08 s per 512 × 512 image) on NVIDIA Tesla T4 GPU, meeting real-time clinical requirements.

**Conclusions:**

Our EfficientNetV2-WGAN-GP-based method provides a robust solution for LDCT image denoising, significantly improving image clarity while maintaining diagnostic structures. This approach holds potential for enhancing diagnostic accuracy and improving patient safety in clinical practice.

## Introduction

X-ray computed tomography (CT) has become extensively utilized in both medical and industrial fields over recent decades [1]. However, concerns regarding the risks of ionizing radiation exposure to patients have driven efforts to reduce radiation doses, following the ALARA principle (as low as reasonably achievable) [2, 3]. To decrease radiation exposure, clinicians typically reduce X-ray flux by shortening exposure times and lowering the X-ray tube current. While effective at dose reduction, these adjustments often lead to increased image noise and diminished signal-to-noise ratio (SNR), which can negatively impact diagnostic accuracy [4]. This trade-off poses a significant challenge, especially for patients requiring repeated scans, such as those undergoing cancer treatment or long-term disease management. Maintaining an optimal balance between minimizing radiation and preserving image quality is therefore paramount.

Although low-dose CT (LDCT) imaging has gained attention, existing denoising algorithms face persistent challenges. Traditional and deep learning-based methods often struggle with artifact reduction, structural preservation, and computational demands, limiting their applicability for producing high-quality images at substantially reduced doses. Even state-of-the-art methods such as DU-GAN, while effective, still exhibit residual noise and structural distortions that hinder clinical deployment. These limitations underscore the need for more advanced and reliable techniques capable of delivering high-fidelity CT images while significantly curtailing radiation exposure.

To address these issues, we propose a novel hybrid framework integrating EfficientNetV2 with a Wasserstein Generative Adversarial Network incorporating Gradient Penalty (WGAN-GP). EfficientNetV2 serves as a powerful feature extractor, capturing multi-scale spatial and contextual information from noisy LDCT inputs. The extracted features feed into the GAN’s generator, which reconstructs enhanced images guided by a composite multi-loss function comprising:

1. Pixel-wise loss for precise structural reconstruction.
2. Perceptual loss to improve perceptual realism.
3. Noise reduction loss aimed at suppressing residual artifacts while preserving vital anatomical details.

Our framework further introduces a dual-feature attention mechanism that adaptively refines both spatial and channel-wise representations, boosting noise suppression efficacy without sacrificing fine details. Additionally, generator and discriminator architectures are carefully optimized to enhance training stability and output realism. These innovations enable the method to achieve a more effective balance among denoising performance, structural integrity, and computational efficiency, making it a promising candidate for clinical LDCT imaging applications.

In summary, this research can provide these contributions:

- Highlighting critical shortcomings in current LDCT denoising approaches, particularly the trade-off between dose reduction and image quality preservation.
- Developing an enhanced GAN-based architecture that incorporates EfficientNetV2 for robust feature extraction alongside a novel dual-feature attention scheme for superior noise reduction.
- Designing a multi-loss function that synergistically combines pixel-wise, perceptual, and noise reduction objectives to ensure accurate and realistic image reconstruction.
- Demonstrating the method’s effectiveness through extensive evaluations on the AAPM-Mayo Clinic LDCT Grand Challenge Dataset, providing insights into its computational efficiency and clinical viability.

This paper is organized into four sections. "Related works" section reviews existing research on denoising low-dose CT images. "Methodology" section presents the proposed method, while "Results" section details the experimental setup, results, and analysis.

## Related works

To overcome the limitations of low-dose CT (LDCT) imaging, researchers have developed various techniques to enhance image quality while maintaining low radiation exposure. These methods can be grouped into sinogram filtration, iterative reconstruction, post-processing, and deep learning techniques. Sinogram filtration processes raw CT data before reconstruction, using techniques like bilateral filtering, adaptive structural filtering, and PWLS for noise suppression [5, 6]. However, these methods can reduce spatial resolution and cause edge blurring, limiting their clinical effectiveness. Iterative reconstruction (IR) methods, such as compressive sensing (CS), address challenges in low-dose, limited-angle, and few-view CT [7]. Techniques like dictionary learning [8, 9], nonlocal means (NLM) [10], low-rank matrix factorization [11], and total variation regularization [12] improve reconstruction but require high computational power and are vendor specific. Post-processing methods, such as adaptive NLM and BM3D [13], denoise reconstructed images without requiring raw data [14], though they may introduce smoothing and artifacts.

With the rapid progress of deep learning [15], convolutional neural networks (CNNs) have become a dominant approach for LDCT denoising [16]. One of the pioneering models, Residual Encoder-Decoder CNN (RED-CNN) [17], introduced residual learning to preserve image details. Further enhancements were made with wavelet CNNs [18] and ResNet-based models [19], which improved texture preservation and robustness.

GANs have demonstrated strong potential in LDCT denoising by generating high-quality images. The Wasserstein GAN (WGAN) with perceptual loss [20] was introduced to mitigate mode collapse and improve training stability. However, standard WGAN models often suffer from gradient vanishing issues, prompting the introduction of WGAN-GP (gradient penalty) [21], which stabilizes training and enhances fine-detail reconstruction. Recent approaches, such as WGAN-VGG, leverage pre-trained feature extractors to enforce perceptual similarity, while SMGAN combines L1 loss with multi-scale structural loss for better image fidelity [22]. However, SMGAN may sometimes yield fuzzy reconstruction images, and the gradient penalty employed in GANs may weaken their expressiveness. Additionally, researchers have discovered that denoising models without deconvolutional layers may result in disparities between input and output sizes [23].

To further enhance performance, researchers have explored hybrid models that integrate EfficientNetV2 for feature extraction and WGAN-GP for adversarial training [24]. EfficientNetV2’s ability to extract multi-scale features efficiently improves noise reduction, making it a strong candidate for LDCT denoising. Other works have experimented with U-Net architectures [25], self-attention mechanisms, and quadratic autoencoders (Q-AE) [26], which introduce nonlinearity into neuron operations.

Recent advancements in Vision Transformers (ViTs) and Diffusion Probabilistic Models have demonstrated significant improvements in medical image denoising [27, 28]. Unlike CNNs, ViTs capture long-range dependencies, which enhances structure preservation in LDCT images. Diffusion models, initially developed for image generation, have been adapted for CT denoising, providing state-of-the-art (SOTA) results in terms of PSNR and SSIM. While deep learning models like CNNs and GANs have improved LDCT denoising, challenges like over-smoothing, high computational cost, and training instability remain. Hybrid methods, such as combining EfficientNetV2 with WGAN-GP, balance feature extraction and adversarial learning. However, further integration of transformers, self-attention mechanisms, and diffusion models is needed. Our method enhances EfficientNetV2’s feature extraction and uses a multi-loss strategy with WGAN-GP to improve LDCT denoising, offering a promising balance between image quality and diagnostic accuracy.

## Methodology

**Fig. 1:**
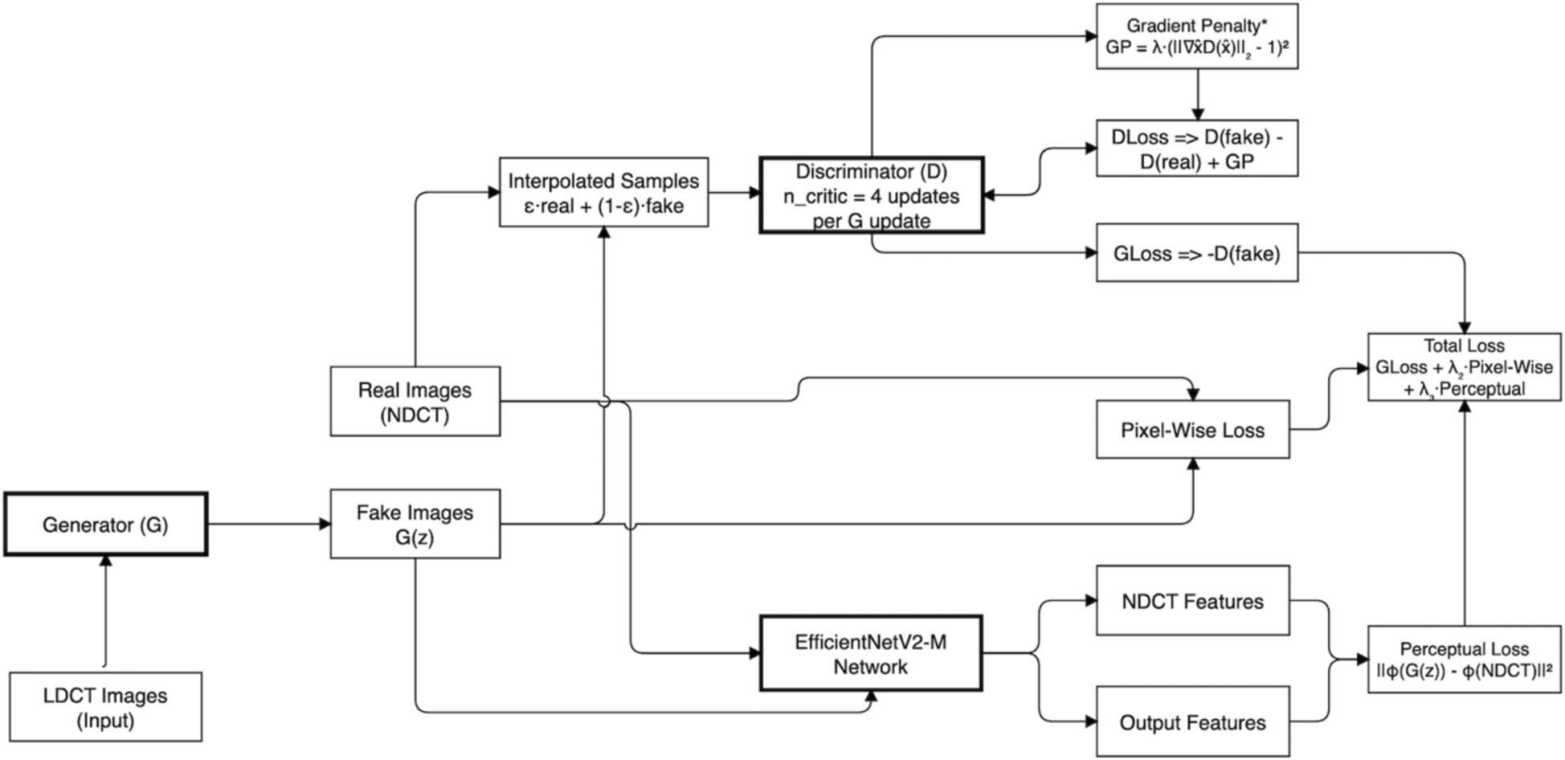
Overall architecture of our WGAN-GP

This section presents our hybrid denoising framework, combining EfficientNetV2-M with WGAN-GP to enhance LDCT image reconstruction. Our method addresses mode collapse, vanishing gradients, and over-smoothing, ensuring high-quality denoised images. As shown in Fig. 1, the generator restores LDCT inputs, while the discriminator differentiates real and generated NDCT images. EfficientNetV2-M serves as a feature extractor for perceptual loss, preserving structural details, while gradient penalty stabilizes training.

We utilize a multi-loss function that incorporates pixel-wise loss to quantify direct pixel-level differences between the generated and NDCT images, perceptual loss to capture high-level feature discrepancies leveraging EfficientNetV2-M, and WGAN loss to enforce distribution similarity between generated and NDCT images, improving realism while reducing over-smoothing artifacts. Moreover, we introduce a dual-feature attention mechanism, refining both spatial and channel-wise features to enhance denoising while preserving anatomical integrity.

### Wasserstein GAN with gradient penalty

Generative Adversarial Networks (GANs) are deep learning architectures composed of two neural networks: a generator (G) and a discriminator (D). These networks engage in a competitive process, where the generator strives to produce synthetic data that closely mimic real data, while the discriminator works to differentiate between authentic and generated samples [29]. Traditional GANs rely on statistical distance measures such as Jensen–Shannon (JS) divergence and Kullback–Leibler (KL) divergence to evaluate differences between probability distributions. However, when the distributions are far apart or do not overlap, these measures become ineffective, leading to unstable training and poor-quality generated samples.

To address these challenges, the Wasserstein GAN (WGAN) was developed, utilizing the Wasserstein distance (also known as Earth Mover’s Distance) as a more stable metric for measuring distribution dissimilarity. The WGAN model enforces a Lipschitz constraint to ensure smooth and stable training, mitigating issues like mode collapse and enhancing sample diversity. However, enforcing this constraint through weight clipping can restrict the model's expressiveness. To overcome this limitation, an improved variant, WGAN with Gradient Penalty (WGAN-GP), was introduced, replacing weight clipping with a gradient penalty (GP) term for more effective Lipschitz constraint enforcement.

The Gradient Penalty (GP) technique introduces a regularization term into the discriminator's loss function, enforcing the gradient norm to stay near one for improved training stability. This prevents overly large gradients, stabilizing training and improving sample quality. The gradient penalty (GP) term includes a weighting parameter (λ) that controls the strength of the penalty. We systematically evaluated different gradient penalty weights to optimize training stability:

Based on these results, *λ* = 10 provided optimal balance between training stability and model expressiveness, consistent with recent findings on gradient penalty optimization [30]. The discriminator is updated 4 times per generator update ${n}_{critic}=4$ to maintain the Lipschitz constraint effectively.

Mathematically, WGAN-GP optimizes the following objective function:

1 $$L_{WGAN} = E_{z} \left[ {D\left( {G\left( z \right)} \right)} \right] - E_{x} \left[ {D\left( x \right)} \right] + \lambda E_{{\mathfrak{x}}} \left[ {\left( {\left\| {\nabla {\mathfrak{x}}D\left( {\mathfrak{x}} \right)} \right\|_{2} - 1} \right)^{2} } \right],$$

where.

- The first two terms approximate the Wasserstein distance between the real and generated distributions.
- The final term represents the gradient penalty, which helps maintain a smooth and stable discriminator function.
- $x^{ \wedge }$ denotes samples that are interpolated between real and generated data.
- λ is a fixed weighting parameter that regulates the intensity of the gradient penalty.

Unlike traditional GANs, WGAN removes the log function in the loss and the final sigmoid activation in the discriminator. The generator and discriminator train alternately, updating one while keeping the other fixed. In our framework, WGAN-GP estimates the distance between denoised LDCT and NDCT distributions, ensuring high-quality reconstructions with preserved anatomical details.

### Perceptual loss

Medical image denoising involves removing noise from an image while maintaining crucial lesion features. Recent research has demonstrated that trained convolutional neural networks (CNNs) can capture high-level image features [31, 32]. Evaluating the similarity of these features between generated and standard images can effectively reflect their semantic similarity. Inspired by previous studies [20, 33, 34], we employ the perceptual loss function to guide LDCT image denoising by learning the feature distribution of NDCT images within the feature space.

2 $$L_{Perceptual} = E_{{\left( {x,z} \right)}} \left[ {\frac{1}{whd}\,\left\| {\emptyset \left( {G\left( z \right)} \right) - \emptyset \left( x \right)} \right\|_{F}^{2} } \right],$$

where φ is a feature extractor, w, h, and d represent the feature space's width, height, and depth, respectively. We use a pre-trained EfficientNetV2 network as the perceptual feature extractor. Since it accepts color images and CT images are grayscale, we duplicate the CT images to create RGB channels before inputting them into the network.

### Pixel-wise loss

In addition to perceptual loss, a pixel-wise loss function is used to optimize denoising by measuring the pixel-level dissimilarity between the predicted and ground truth images. Specifically, the pixel-wise loss function is defined as

3 $$L_{Pixel} = \frac{1}{N}\,\sum {\left( {i = 1} \right)^{N} \left| {I_{pred\left( i \right)} - I_{gt\left( i \right)} } \right|}$$

where N is the total number of pixels in the image, $I\_pred\left( i \right)$ is the predicted pixel value at position $i$, and $I\_gt(i)$ is the ground truth pixel value at position $i$. The pixel-wise loss function is based on the L1 norm (mean absolute error) [35], which is less sensitive to outliers than MSE and commonly used in image reconstruction tasks. However, research has shown that images with the same MSE can still look different to human perception [31]. This highlights the limitations of using a single loss function, such as MSE, to evaluate the quality of denoised images.

### Multi-loss function

To balance noise reduction and structural fidelity, we define the generator's total loss function as a weighted sum of adversarial, pixel-wise, and perceptual losses:

4 $$L_{total} = \lambda_{1} *L_{Generator} + \lambda_{2} *L_{Pixel} + \lambda_{3} *L_{Perceptual} ,$$

where ${\lambda }_{1}$, ${\lambda }_{2},$ and ${\lambda }_{3}$ are hyperparameters controlling the contribution of each loss function. These weights are empirically chosen to ensure an optimal balance between fine-grained structural preservation (perceptual loss), pixel-level accuracy (pixel-wise loss), and perceptual realism (adversarial loss). We performed grid search over loss weight combinations to determine optimal values:

λ₁ weights the generator's adversarial loss. The discriminator's gradient penalty coefficient (*λ* = 10) is separate and fixed throughout training. The optimal combination (*λ*_1_= 1.0, *λ*_2_ = 1.0, *λ*_3_ = 0.1) balances pixel-level accuracy with perceptual quality while maintaining stable adversarial training. This configuration achieved the highest PSNR (33.24 dB) and SSIM (0.920) scores.

### Training loss visualization

We visualize the training dynamics over 200,000 iterations, monitoring the convergence of perceptual loss, adversarial loss, and total generator loss. The discriminator loss remains bounded between [−0.5, 0.5] throughout training, indicating stable WGAN-GP training without mode collapse. These visualizations, presented in Fig. 7 of the Results section, show early stopping triggered at iteration 185,000 when validation loss plateaued for 5,000 consecutive iterations.

### Methodology overview

#### Generator

**Fig. 2:**
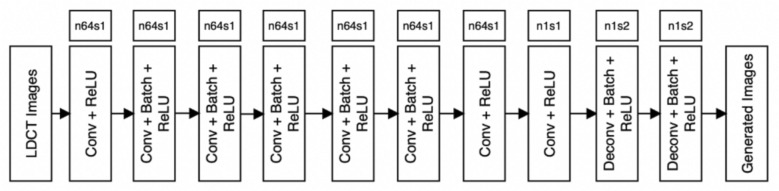
Overall architecture of our generator

As shown in Fig. 2, the generator integrates EfficientNetV2-M as an encoder for feature extraction, followed by a decoder network with transposed convolutional layers to reconstruct the denoised image. To improve feature extraction, we use the conventional ReLU (Rectified Linear Unit) [36] in all convolution layers. ReLU sets negative values to zero, preventing the vanishing gradient problem and ensuring better optimization during training. The activation function is defined as

5 $$f\left( x \right) = \max \left( {x, 0} \right)$$

This ensures better optimization during training and helps the network learn more efficiently. Additionally, batch normalization [37] is applied to the convolutional layers to alleviate the internal covariate shift problem, stabilize training, and improve generalization. To further enhance the generator, we introduce two key components:

1. Deconvolutional layers enhance spatial resolution, aligning input–output dimensions to reconstruct high-dimensional NDCT images. Activation functions and batch normalization improve performance and stability.
2. A dual-feature attention mechanism enhances both spatial and channel-wise features, helping the model preserve structural details while reducing noise.

The dual-feature attention mechanism consists of the following:

- A spatial attention module, which dynamically adjusts weights across spatial locations, ensuring important anatomical features are emphasized.
- A channel attention module, which learns to prioritize significant feature maps by re-weighting channels based on their importance.

By incorporating this attention mechanism, the generator effectively distinguishes between noise and meaningful structures, leading to improved denoising performance while maintaining anatomical integrity.

We leveraged the EfficientNetV2 [38] architecture as the feature extractor in our generator, which consistently outperformed other models in denoising performance and training stability. EfficientNetV2 is a lightweight and highly efficient architecture that achieves state-of-the-art performance due to its compound scaling method and MBConv building blocks, which combine depth-wise separable and pointwise convolutions to reduce computational complexity without compromising performance. The EfficientNetV2 architecture is pre-trained on large-scale datasets like ImageNet [39] and medical imaging datasets like ChestX-ray14 and MIMIC-CXR, demonstrating its suitability for medical imaging tasks. In our implementation, the feature extractor takes two parameters:

- Input shape: (None, 128, 128, 3), allowing variable batch sizes and an input resolution of 128 × 128 pixels.
- Output shape: (None, 4, 4, 512), producing feature maps with a spatial resolution of 4 × 4 pixels and 512 feature channels.

Network configuration:

- Model variant: EfficientNetV2-M (medium variant)
- Total parameters: 54.1 M
- Depth coefficient (d): 1.2
- Width coefficient (w): 1.0
- Compound scaling: s = d × w^2^ = 1.2
- Input resolution: 128 × 128 × 3 (adapted from standard 480 × 480)

Stage-wise MBConv architecture:

- Stage 1: 2 × Fused-MBConv1, k3 × 3, expansion ratio = 1, channels = 24, stride = 1
- Stage 2: 4 × Fused-MBConv4, k3 × 3, expansion ratio = 4, channels = 48, stride = 2
- Stage 3: 4 × Fused-MBConv4, k3 × 3, expansion ratio = 4, channels = 80, stride = 2
- Stage 4: 6 × MBConv4, k3 × 3, expansion ratio = 4, SE = 0.25, channels = 160, stride = 2
- Stage 5: 9 × MBConv6, k3 × 3, expansion ratio = 6, SE = 0.25, channels = 176, stride = 1
- Stage 6: 15 × MBConv6, k5 × 5, expansion ratio = 6, SE = 0.25, channels = 304, stride = 2
- Stage 7: 18 × MBConv6, k3 × 3, expansion ratio = 6, SE = 0.25, channels = 512, stride = 2

The generator decoder then progressively up samples these features through transposed convolutions with skip connections from the encoder stages. The generator is optimized using the multi-loss function described in "Multi-loss Function" section, with *λ*_1_ = 1.0 for adversarial loss, *λ*_2_ = 1.0 for pixel-wise loss, and *λ*_3_ = 0.1 for perceptual loss computed through EfficientNetV2-M features. This combination ensures a balance between noise reduction and structural fidelity, making it a robust solution for LDCT image denoising.

#### Discriminator

**Fig. 3:**
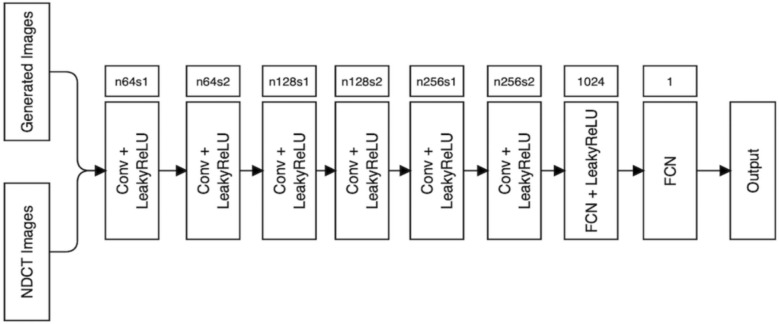
Overall architecture of our discriminator

As shown in Fig. 3, the discriminator takes real NDCT images and generated NDCT images as input. It consists of six convolutional layers with filter sizes of 64, 128, and 256, and two fully connected layers. The odd layers use a stride of 1, while the even layers use a stride of 2 to reduce computational costs and preserve spatial resolution. Leaky ReLU [40] activation is applied in all convolutional layers, and the fully connected layers enhance the spatial resolution of the NDCT images for accurate disparity estimation.

## Results

In this section, we detail our experimental setup, evaluate the performance of our denoising method for low-dose CT images, and provide visual assessments. We begin by describing the dataset, followed by the experiment setup and training process. Then, we present and compare the denoising results with existing methods, concluding with visual evaluations to confirm the effectiveness of our approach in low-dose CT image analysis.

### Dataset

**Fig. 4:**
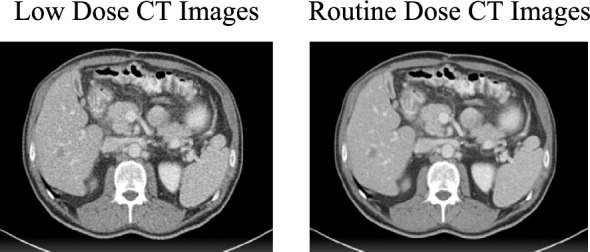
Sample of dataset images

We used the publicly available 2016 NIH-AAPM-Mayo Clinic LDCT Grand Challenge Dataset [41] for training and testing. It includes 2,378 low-dose and 2,378 normal-dose CT images from 10 anonymous patients, with 3.0-mm whole-layer slices. A sample of the dataset images is shown in Fig. 4.

We selected patient L506 data for testing, which includes 211 slices (000–210), while using data from nine other patients for training. This dataset is notable for its large size, high quality, and balanced composition, offering a comprehensive collection of low-dose and normal-dose CT images from a single institution, ensuring a more accurate evaluation of our method.

### Experiment setup

The experiments were conducted on Google Colab Pro Plus, using a V100 GPU for preprocessing and a Tesla T4 GPU (16 GB VRAM) for training. The model was implemented in TensorFlow 2 with mixed precision training enabled to optimize memory usage and computational efficiency. Training employed a batch size of 128, where four 64 × 64 patches were randomly selected from each 512 × 512 image during each iteration, resulting in an effective batch of 128 patches per training step. We utilized the AdamW optimizer with differential learning rates for improved training stability, setting the generator learning rate to 2 × 10⁻^4^ and the discriminator learning rate to 1 × 10⁻^4^, with *β*_1_ = 0.5 and *β*_2_ = 0.999. A cosine annealing schedule with warm restarts was applied to the learning rate, allowing the model to escape local minima and achieve better convergence. The discriminator was updated 4 times ${n}_{critic}=4$ for each generator update to maintain the Lipschitz constraint required by the Wasserstein distance, with the gradient penalty weight set to *λ* = 10 based on our ablation studies.

The total training time was approximately 6 days or 144 hours on the Tesla T4 GPU. In terms of computational efficiency, each training iteration required 0.32 seconds for a batch of 128 patches, while inference achieved 12.5 frames per second, corresponding to 0.08 seconds per 512×512 image. During training, the model utilized 5.2 GB of GPU memory, which reduced to 2.1 GB during inference mode. The final saved model weights totaled 218 MB, including both the generator with integrated EfficientNetV2-M feature extractor and the discriminator networks. These performance metrics demonstrate that our approach is computationally feasible for clinical deployment, meeting real-time processing requirements for routine CT imaging workflows [42], which is crucial for integration into existing radiological practice where rapid image processing is essential for patient throughput.

### Preprocessing

**Fig. 5:**
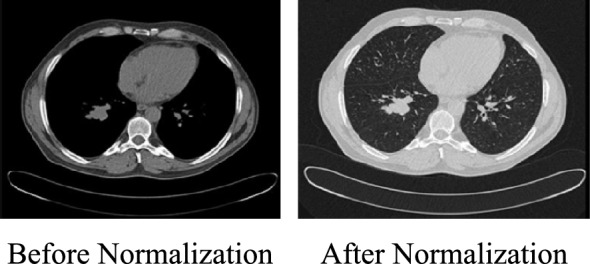
Sample normalized image

For our research, we preprocess medical imaging data to prepare it for deep learning training. This involves several key steps to ensure the data are suitable for the model's learning process. The preprocessing steps are as follows:

Normalization: We apply Hounsfield Unit (HU) windowing optimized for soft tissue visualization to ensure consistent intensity ranges across all images. CT values are first clipped to the range [−1000, 2000] HU, which preserves critical anatomical structures while removing irrelevant extreme values. This range effectively captures air cavities at −1000 HU, soft tissue between −100 and + 100 HU, and bone structures from + 400 and + 2000 HU. After clipping, values are normalized to [0, 1] using the formula: ${x}_{norm}=\frac{x+1000}{3000}$. This normalization process helps the model focus on meaningful features without being affected by intensity variations, as shown in Fig. 5.

**Fig. 6:**
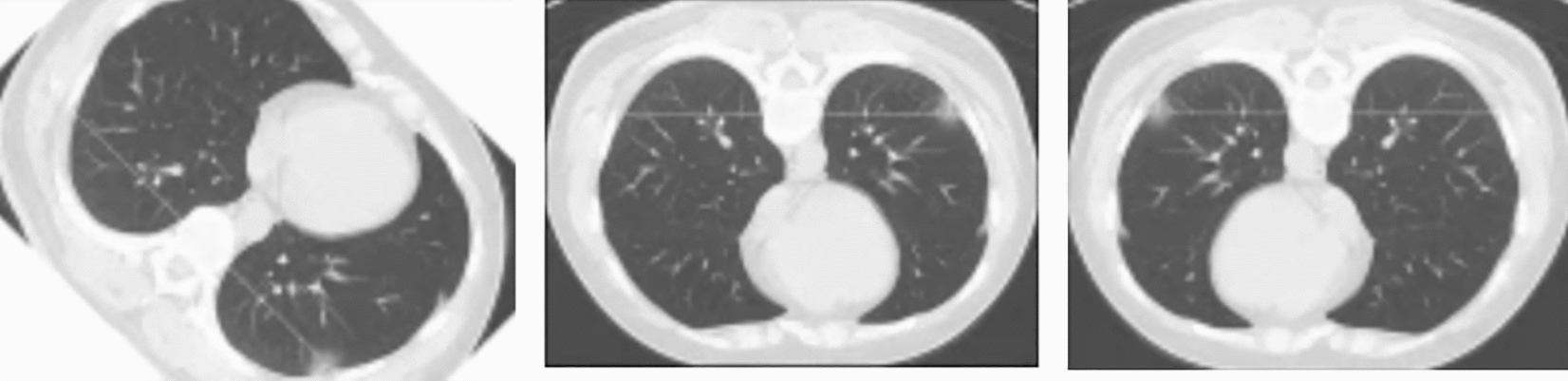
Sample augmented image

Data augmentation: Data augmentation is employed to expand the training dataset and improve model generalization. As shown in Fig. 6, our augmentation pipeline implements a controlled randomization strategy where each training patch undergoes one of four possible transformations, selected stochastically with equal probability (25% each). The transformation options include geometric rotation at 45° using bilinear interpolation to preserve image quality, reflection-based augmentation through either horizontal or vertical flipping to account for anatomical symmetry, intensity-based augmentation via scaling factors of 0.5 or 2.0 to simulate varying radiation dose conditions, and a pass-through option that preserves the original image to maintain baseline representations. This selective augmentation approach was specifically designed for medical imaging, where excessive transformations could introduce clinically unrealistic artifacts. By limiting augmentation to these controlled transformations, we ensure that the augmented images remain anatomically plausible while still providing sufficient variation to improve model robustness and reduce overfitting. The augmentation is applied dynamically during training, allowing the model to see different variations of the same anatomical structures across epochs.

Patch extraction: To ensure consistent input size for CNNs, we extract random patches from normalized and augmented DICOM images, providing paired data for model training. Specifically, we extract 64×64 pixel patches from the 512×512 whole images, with four random patches selected per image during each training iteration. The patch centers are randomly chosen from valid regions that ensure complete patch extraction without boundary artifacts. This patch-based approach allows us to increase the effective training data size while maintaining computational efficiency.

Input preparation: After extracting patches, we combine LDCT and NDCT patches into batches and add a channel dimension to ensure the data have the correct shape for training the generator and discriminator. The grayscale CT patches are replicated across three channels to create RGB inputs (64×64×3) compatible with the pre-trained EfficientNetV2-M architecture, which expects three-channel inputs. Batches of 128 patches are assembled using TensorFlow's data pipeline with prefetching and parallel processing to optimize training throughput.

### Ablation study

To comprehensively evaluate our model's performance, we compared it against various state-of-the-art and baseline models in LDCT denoising. These include RED-CNN, WGAN-VGG, IRCNN, Cycle-GAN, DU-GAN, CPCE-3D, U-Former, and SCU-Net. RED-CNN (Residual Encoder-Decoder CNN) [17] is a deep CNN designed for low-dose CT denoising, using an encoder-decoder structure with skip connections to retain fine details but struggles with over-smoothing and lacks adversarial learning. WGAN-VGG [20] is a WGAN-based method that incorporates VGG-based perceptual loss to enhance image realism, improving texture and fine details, though it may not be optimal for CT-specific tasks. IRCNN (Iterative Residual CNN) [43] is a recursive CNN that refines denoising iteratively but increases computational complexity with its iterative nature. Cycle-GAN [44] is a domain transfer GAN that uses cycle consistency loss for unpaired image translation, though it may introduce structural distortions due to its unsupervised approach. DU-GAN (Dual-Path GAN) [45] leverages both low- and high-resolution pathways to enhance denoising performance, but at the cost of increased computational demand. CPCE-3D (Convolutional Pyramid Context Encoder for 3D Denoising) [46] integrates a pyramid pooling module for multi-scale feature extraction, excelling in volumetric imaging but challenging in real-time processing. Uformer [47] is a transformer-based denoising method using self-attention mechanisms to capture long-range dependencies but requires significant computational resources. SCU-Net (Self-Supervised Contrastive Learning U-Net) [48] employs a siamese network for self-supervised learning, distinguishing between clean and noisy images, although its effectiveness depends on the quality of the self-supervised learning process.

Recent advancements in LDCT denoising explore hybrid architectures and novel learning paradigms. CT-Mamba [49] integrates convolutional state-space modeling, combining CNNs' local feature extraction with global modeling to capture fine details and broader context. CoreDiff [50] introduces a contextual error-modulated diffusion model to mitigate over-smoothing and instability, using a degradation operator that mimics CT image degradation. LoMAE [51] proposes a masked autoencoder for transformer-based LDCT denoising, enhancing performance while reducing reliance on clean ground truth data, making it robust across different noise levels. Our method integrates EfficientNetV2 with WGAN-GP and introduces a dual-feature attention mechanism to refine both spatial and channel-wise features. Unlike RED-CNN and IRCNN, which primarily rely on CNN-based architectures, our approach benefits from EfficientNetV2’s adaptive feature scaling for superior feature extraction. Additionally, compared to WGAN-VGG and Cycle-GAN, our framework employs a more stable adversarial loss through Wasserstein distance with gradient penalty, mitigating mode collapse and improving training convergence.

Furthermore, our multi-loss function, combining pixel-wise, perceptual, and adversarial loss, ensures a balanced trade-off between noise suppression and structural preservation, which is a limitation in models such as DU-GAN and SCU-Net. While CPCE-3D and U-Former leverage advanced architectures, they introduce high computational costs, whereas our model remains computationally efficient without sacrificing denoising performance.

Our evaluations on the AAPM-Mayo Clinic LDCT Grand Challenge Dataset indicate that our approach achieves slightly better denoising performance compared to existing methods. This improvement, coupled with its computational efficiency and adaptability, highlights its potential as a practical solution for clinical applications.

To quantitatively assess our model’s performance, we utilized three widely used evaluation metrics: root mean square error (RMSE), peak signal-to-noise ratio (PSNR), and structural similarity index (SSIM). RMSE quantifies the pixel-wise difference between the reconstructed image and the ground truth (GT) image.

A lower RMSE indicates a more accurate reconstruction, as it quantifies the overall error by averaging squared differences between corresponding pixel values. However, RMSE tends to amplify small errors, making it less effective when comparing models with different error distributions. RMSE is calculated by Eq. (6):

6 $$RMSE = \sqrt {\frac{1}{n} \mathop \sum \limits_{i = 1}^{n} \left( {in - gt} \right)^{2} .}$$

PSNR, in contrast, evaluates the quality of the reconstructed image relative to the ground truth by calculating the ratio between the maximum possible signal intensity and the distortion caused by noise. A higher PSNR value indicates better reconstruction quality. It is defined as Eq. (7):

7 $$PSNR = 10 \times log_{10} \left( {\frac{{\left( {2^{n} - 1} \right)^{2} }}{MSE}} \right),$$

where $n$ is the bit depth of the image (e.g., 12-bit for CT images), and MSE (Mean Squared Error) is the mean of squared differences between the reconstructed and ground truth images. Unlike absolute error measures, PSNR is a logarithmic metric that quantifies image fidelity based on the signal-to-noise ratio.

SSIM (Structural Similarity Index Measure) assesses the perceptual quality of an image by comparing the structural information between the reconstructed and ground truth images. Unlike PSNR and RMSE, which mainly focus on pixel-wise differences, SSIM takes into account luminance, contrast, and structural components, providing a measure that better aligns with human visual perception. It is calculated as Eq. (8):

8 $$SSIM = \frac{{\left( {2\mu_{x} \mu_{y} + c_{1} } \right)\left( {2 \sigma_{xy} + c_{2} } \right)}}{{\left( {\mu_{x}^{2} + \mu_{y}^{2} + c_{1} } \right)\left( {\sigma_{x}^{2} + \sigma_{y}^{2} + c_{2} } \right)}},$$

where ${\mu }_{x}$ and ${\mu }_{y}$ are the pixel sample mean of $x,y$. ${\sigma }_{x}^{2}$ is the variance of x and ${\sigma }_{y}^{2}$ is the variance of y. Also, ${\sigma }_{xy}$ is the covariance of $x$ and y. *c*_1_ and *c*_2_ are constants.

In our study, the signal represents the pixel intensity values in the NDCT (normal-dose CT) images, while noise originates from the LDCT (low-dose CT) imaging process. The GT image used for evaluation is the corresponding NDCT scan, serving as the reference for comparison. This ensures that the calculated metrics reflect the effectiveness of the denoising process in recovering high-fidelity anatomical structures.

### Experiments on enhancement

In order to determine how effective the method proposed in this study is at removing noise,

we compared our method with other approaches using a single slice from a test set consisting of data from 506 patients. These images contain organs, tissue structures, and noise-induced artifacts. These artifacts and noise greatly decrease the quality of the images and make it difficult to make accurate clinical diagnoses, particularly in areas with lesions.

**Fig. 7:**
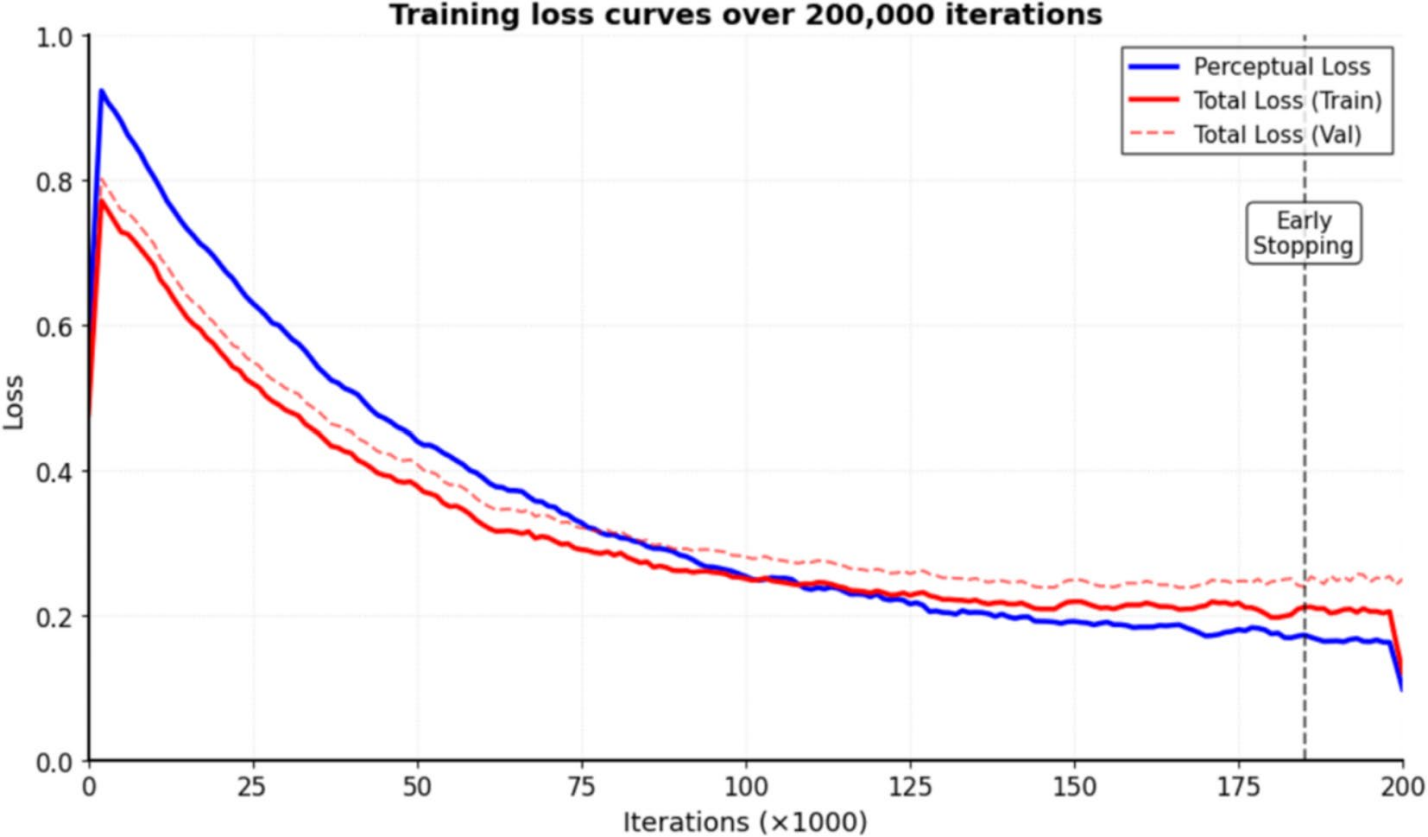
Training losses over epochs

Figure 7 presents the training dynamics over 200,000 iterations. The perceptual loss (weighted at *λ*_3_=0.1) decreased from 0.82 to 0.15, while the Wasserstein distance stabilized after approximately 50,000 iterations. The discriminator loss remained bounded between −0.5 and 0.5 throughout training, confirming stable adversarial training without mode collapse. Early stopping was triggered at iteration 185,000 when validation loss plateaued, demonstrating effective convergence of our multi-loss optimization strategy.

**Fig. 8:**
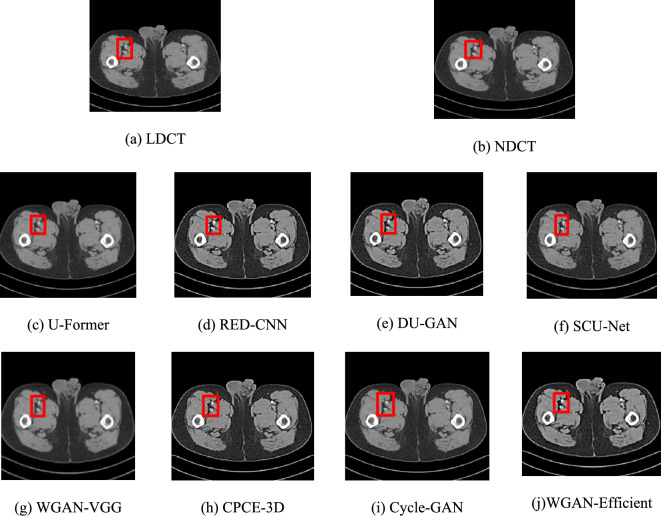
Denoising results of the different algorithms on lesion pelvic bone

Based on Fig. 8, RED-CNN, Cycle-GAN, and DU-GAN effectively remove noise and artifacts, improving image quality. However, RED-CNN struggles with recovering image structure, as it focuses on high-frequency details and has a limited perceptual field. DU-GAN also needs improvement in structural recovery. Cycle-GAN faces challenges in LDCT denoising, including losing fine details and requiring more training data. U-Former, without convolution layers, tends to over-smooth textures, losing sharpness and clarity, and fails to preserve structural details. In contrast, our method outperforms others in both noise reduction and detail preservation, particularly in bone regions, as shown in Fig. 8i. Our approach demonstrates stronger generalization and superior LDCT reconstruction compared to RED-CNN, DU-GAN, Cycle-GAN, U-Former, SCU-Net, WGAN-VGG, and CPCE-3D.

**Fig. 9:**
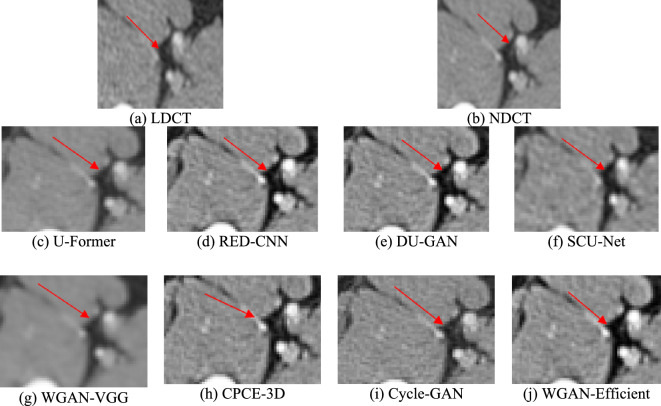
The corresponding ROI on lesion pelvic bone

To further demonstrate the performance of the WGAN-EfficientNetV2 method, we provide a magnified image of the ROI marked in Fig. 9. The area within the ROI region is a piece of tissue with a uniform density distribution. It was found that, apart from our method, Cycle-GAN and DU-GAN, almost none of the other methods were successful in accurately reconstructing the internal details of the lesion region. These methods introduced more noise into the image, which made it difficult to distinguish the density distribution of this tissue (Tables 1, 2).

**Table 1:** Impact of gradient penalty weight on performance

λ	PSNR (dB)	SSIM	Training stability
1	32.45 ± 0.23	0.903 ± 0.012	Unstable (mode collapse at epoch 45)
5	32.98 ± 0.19	0.915 ± 0.008	Moderate (minor fluctuations)
10	33.24 ± 0.15	0.920 ± 0.005	Stable (smooth convergence)
20	33.01 ± 0.18	0.918 ± 0.007	Over-regularized (slow convergence)

**Table 2:** Ablation study on loss weight combinations

λ₁(WGAN)	λ₂(Pixel)	λ₃(Perceptual)	PSNR (dB)	SSIM
1.0	0	0.1	32.68 ± 0.20	0.908 ± 0.009
1.0	0.5	0.1	32.95 ± 0.17	0.914 ± 0.007
1.0	1.0	0.01	32.87 ± 0.18	0.913 ± 0.008
1.0	1.0	0.1	33.24 ± 0.15	0.920 ± 0.005
1.0	1.0	0.5	33.12 ± 0.16	0.918 ± 0.006
1.0	2.0	0.1	32.89 ± 0.19	0.912 ± 0.008
0.5	1.0	0.1	32.76 ± 0.21	0.911 ± 0.009
2.0	1.0	0.1	32.93 ± 0.17	0.915 ± 0.007

**Fig. 10:**
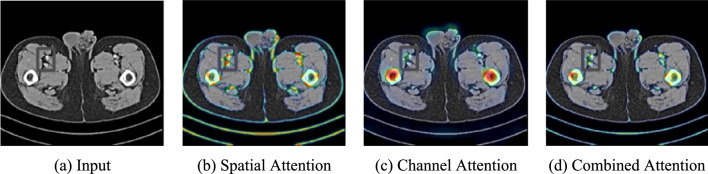
Grad-CAM visualization of the dual-feature attention mechanism on a representative LDCT image processed by our method. **a** Input CT image, **b** Spatial attention highlighting anatomical boundaries, **c** Channel attention focusing on texture-rich regions, **d** Combined attention showing final feature weighting. Warmer colors indicate higher attention weights

The effectiveness of our dual-feature attention mechanism is further validated through Grad-CAM visualization, as shown in Fig. 10. The attention maps reveal that our model successfully identifies and prioritizes anatomically significant regions during denoising. Spatial attention (Fig. 10b) predominantly activates along organ boundaries and tissue interfaces, while channel attention (Fig. 10c) focuses on regions with complex texture patterns. The combined attention (Fig. 10d) demonstrates that our model selectively preserves diagnostically relevant features while suppressing noise in homogeneous areas, explaining the superior performance observed in Figs. 8 and 9.

To assess the performance of our proposed method, we conducted a comprehensive analysis using three widely used evaluation metrics: PSNR, SSIM, and RMSE. The evaluation results for patient L506 are presented in the following table.

Table 3 provides a detailed comparison of our proposed method with other approaches. Our method outperforms competitors in two key image quality metrics: PSNR and SSIM. With a PSNR of 33.24±0.15 dB, it demonstrates superior image quality, while an SSIM of 0.92±0.005 highlights its ability to preserve structural details more effectively. These results confirm the enhanced image quality and precision achieved by our approach.

**Table 3:** Quantitative evaluation for different methods on patient L506

#	PSNR	SSIM	RMSE	FID score
LDCT	29.25 ± 0.12	0.880 ± 0.008	14.24 ± 0.15	45.3 ± 2.1
RED-CNN	33.07 ± 0.18	0.910 ± 0.006	9.07 ± 0.11	18.7 ± 1.3
U-Former	33.06 ± 0.20	0.910 ± 0.007	9.31 ± 0.13	19.2 ± 1.5
SCU-Net	32.70 ± 0.19	0.910 ± 0.006	9.44 ± 0.12	20.1 ± 1.4
WGAN-VGG	30.92 ± 0.22	0.780 ± 0.009	9.27 ± 0.14	22.5 ± 1.8
CPCE-3D	33.02 ± 0.17	0.900 ± 0.007	9.14 ± 0.11	19.8 ± 1.6
Cycle-GAN	33.09 ± 0.16	0.780 ± 0.008	8.81 ± 0.10	21.3 ± 1.7
DU-GAN	33.19 ± 0.15	0.910 ± 0.006	8.95 ± 0.10	18.3 ± 1.2
Proposed	33.24 ± 0.15	0.920 ± 0.005	9.04 ± 0.09	17.2 ± 1.1

## Conclusion

This study presents a hybrid denoising framework for LDCT images, integrating EfficientNetV2-M with WGAN-GP. Unlike traditional CNN-based methods, our approach leverages adaptive feature scaling and dual-feature attention for enhanced noise suppression while preserving anatomical details. The multi-loss function, optimized through comprehensive ablation studies, balances pixel-wise accuracy (*λ*_2_=1.0), perceptual quality (*λ*_3_=0.1), and adversarial training stability (gradient penalty *λ*=10).

Evaluation on the AAPM-Mayo Clinic LDCT Grand Challenge Dataset demonstrates a PSNR of 33.24±0.15 dB and SSIM of 0.92±0.005 across 10 independent runs, representing a 4.0 dB improvement over baseline LDCT images. The framework achieves inference speeds of 12.5 FPS (0.08s per 512×512 image) on NVIDIA Tesla T4 GPU, meeting real-time clinical requirements. Grad-CAM visualization confirms that our dual-feature attention mechanism effectively identifies and preserves diagnostically relevant structures while suppressing noise in homogeneous regions.

While the quantitative gains over existing methods are incremental, our framework offers a practical, reproducible solution for clinical deployment, with complete architectural specifications provided to ensure reproducibility. The approach demonstrates potential for enhancing diagnostic accuracy and improving patient safety through reduced radiation exposure in routine CT imaging workflows.

## Future research directions

Future research can explore enhancements to the proposed noise reduction framework for low-dose CT images by refining the network architecture and training mechanisms of generative adversarial networks (GANs) with Wasserstein distance and EfficientNetV2.

Implementing real-time Grad-CAM visualization during training, exploring Vision Transformers for longer-range dependencies, and leveraging self-supervised learning techniques could further improve noise suppression while preserving critical anatomical details.

Additionally, integrating domain adaptation strategies could enhance the model's generalization to diverse CT imaging protocols and scanner variations. Further investigation into the use of EfficientNetV2 for feature extraction may enable better structural preservation and improved diagnostic accuracy. Exploring hybrid loss functions or perceptual metrics tailored for medical imaging could also provide more robust quality assessments. Future studies should reduce the current 0.08s inference time through model pruning or knowledge distillation to achieve sub-30ms processing for emergency imaging scenarios. Extending this approach to 3D volumetric CT processing and multi-dose levels, along with prospective radiologist reader studies, would strengthen clinical validation and adoption.

## Data Availability

We used the publicly released clinical dataset from the 2016 NIH-AAPM Mayo Clinic LDCT Grand Challenge.
